# Topographical mapping of the mechanical characteristics of the human neurocranium considering the role of individual layers

**DOI:** 10.1038/s41598-020-80548-y

**Published:** 2021-02-12

**Authors:** Johann Zwirner, Sarah Safavi, Mario Scholze, Kai Chun Li, John Neil Waddell, Björn Busse, Benjamin Ondruschka, Niels Hammer

**Affiliations:** 1 Department of Anatomy, University of Otago, Dunedin, New Zealand; 2 Institute of Materials Science and Engineering, Chemnitz University of Technology, Chemnitz, Germany; 3 Institute of Macroscopic and Clinical Anatomy, University of Graz, Graz, Austria; 4 Sir John Walsh Research Institute, University of Otago, Dunedin, New Zealand; 5 Department of Osteology and Biomechanics, University Medical Center Hamburg-Eppendorf, Hamburg, Germany; 6 Institute of Legal Medicine, University Medical Center Hamburg-Eppendorf, Hamburg, Germany; 7 Institute of Legal Medicine, University of Leipzig, Leipzig, Germany; 8 Department of Orthopedic and Trauma Surgery, University of Leipzig, Leipzig, Germany; 9 Fraunhofer IWU, Dresden, Germany

**Keywords:** Bone, Tissues, Anatomy

## Abstract

The site-dependent load-deformation behavior of the human neurocranium and the load dissipation within the three-layered composite is not well understood. This study mechanically investigated 257 human frontal, temporal, parietal and occipital neurocranial bone samples at an age range of 2 to 94 years, using three-point bending tests. Samples were tested as full-thickness three-layered composites, as well as separated with both diploë attached and removed. Right temporal samples were the thinnest samples of all tested regions (median < 5 mm; *p* < 0.001) and withstood lowest failure loads (median < 762 N; *p* < 0.001). Outer tables were thicker and showed higher failure loads (median 2.4 mm; median 264 N) than inner tables (median 1.7 mm, *p* < 0.001; median 132 N, *p* = 0.003). The presence of diploë attached to outer and inner tables led to a significant reduction in bending strength (with diploë: median < 60 MPa; without diploë: median > 90 MPa, *p* < 0.001). Composites (r = 0.243, *p* = 0.011) and inner tables with attached diploë (r = 0.214, *p* = 0.032) revealed positive correlations between sample thickness and age. The three-layered composite is four times more load-resistant compared to the outer table and eight times more compared to the inner table.

## Introduction

A comprehensive understanding of the load-deformation behavior of the human neurocranium is paramount to reliably predict head impact scenarios or injury mechanisms using of computational head models^1–3^. The human neurocranium forms a three-layered composite consisting of two compact tables that enclose the cancellous diploë in a sandwich-like manner^4^. In contemporary finite element models, the diploë is either neglected or represented in an oversimplified manner due to the lacking or controversial material properties that are available in the scientific literature^5^. Previous research regarding the load-deformation behavior of the human neurocranium mainly focused on full-thickness composites^6–17^. Only few studies investigated the two tables^7,18–23^ or even the cancellous diploë layer individually^13,18,24,25^. However, an in-depth investigation that mechanically compares the inner and outer table with and without the adjacent diploë and their relation to the full-thickness three-layered neurocranial composite is missing to date, and, therefore, the contribution of the individual layers to the overall mechanical behavior remains unclear.

Flat bones of the cranial vault considerably vary in thickness, and even within the individual bones along defined axes^17,26,27^. Furthermore, it was shown that the thickness of the neurocranium steadily increases during the first two decades of life^28,29^, which potentially continues up to the age of 60 years^30^, and then decreases later in life^31^. However, the age-related change of the neurocranial thickness after the second decade of life was challenged by other studies^26,32^. A correlation between the load-deformation behavior of the cranial bone and its thickness was observed before^6,17,33,34^ with the latter being linked to the ratio between diploë to the two compact tables^17^. Moreover, the bone thickness was related to the dynamic impact response of the human neurocranium observed using finite element modeling^33^. The diploë of the occipital region was stated to be thicker compared to the other neurocranial bones, however, the occipital bone remained mechanically almost unexplored to date^6,10^.

This given study aimed at comparing the mechanical properties of the complete three-layered neurocranial composite with the individual behavior of the layers after being separated as a pioneering step to gain insight into the morpho-mechanicals of the human cranial vault. Investigating the large flat bones of the human neurocranium in an age range of almost one century will enable to observe the related load dissipation in relation to age, sex, layer thickness and time since death, and thus enhance the understanding of the trabecular impact on the complete bone biomechanics^35^.

## Materials/methods

### Retrieval and processing of human neurocranial samples

**Figure 1:**
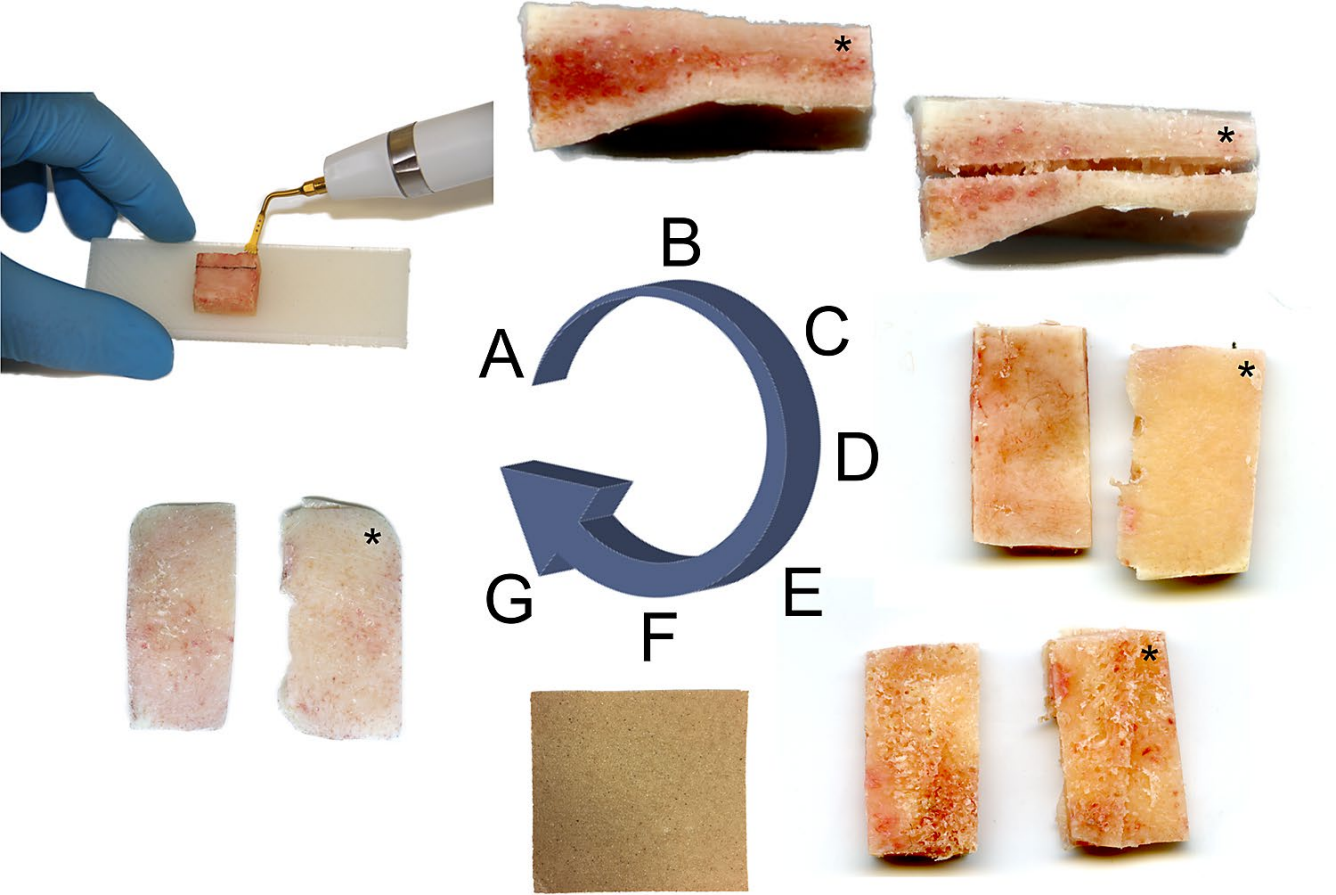
The sample preparation for the mechanical testing is shown. (**A**) Sample cutting using an ultrasound bone cutter, (**B**) Three-layered full-thickness neurocranial composite (“full-thickness composite” group); (**C**) separated outer (*) and inner table (“tables with diploë” group); (**D**) view on surface of the outer (*) and inner table after separation; (**E**) view on diploë-facing side of the outer (*) and inner table after separation; (**F**) sandpaper (60-grit); (**G**) view on outer (*) and inner table according to (**E**) after diploë was removed with sandpaper (“tables without diploë” group).

**Figure 2:**
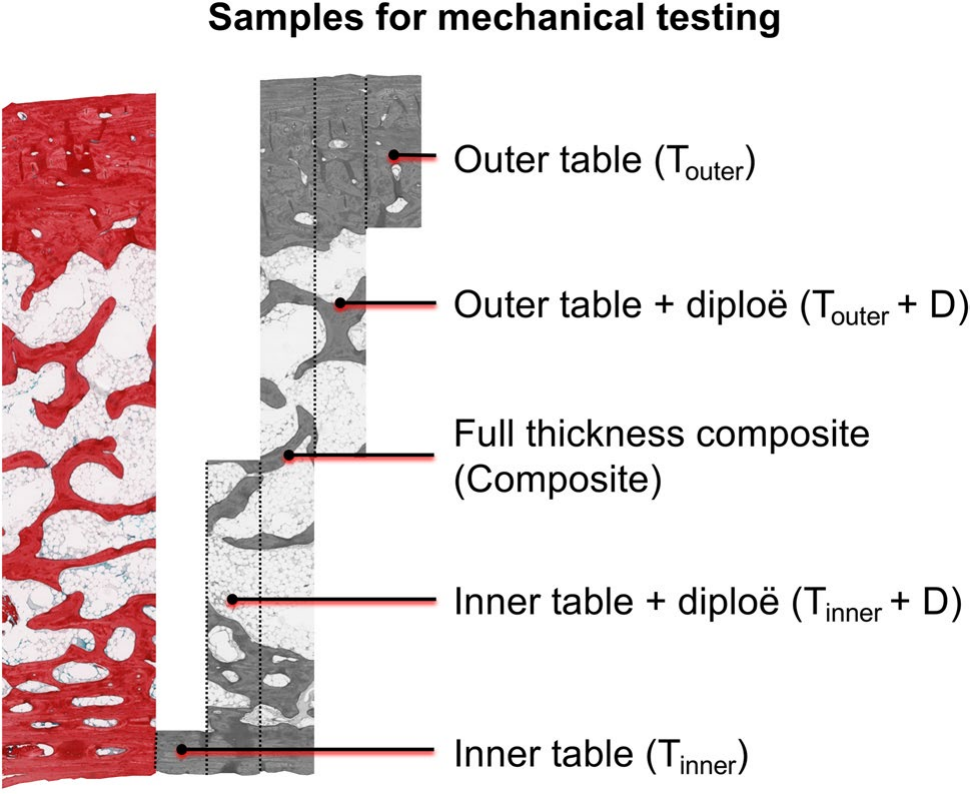
A picrosirius red-stained neurocranial bone sample is depicted to visualize the samples for mechanical testing on a histological level. Full-thickness composite samples formed the “composite” group, the outer and inner tables including the adjacent diploë the “tables with diploë” group and the outer and inner tables without the diploë the “tables without diploë” group.

A total of 257 human neurocranial samples were retrieved from 73 cadavers (25 females, 48 males; age range 2–94 years) during forensic autopsies. Initially, samples of approximately 20 × 20 mm were retrieved from the frontal (n = 60), temporal left (n = 47), temporal right (n = 41), parietal (n = 53) and occipital (n = 56) region. More specifically, samples were retrieved according to the following rules: frontal bone: superior to the orbit at a level between the supraorbital margin and the coronal suture; temporal bone: squamous part; parietal bone: anterior–superior part between the sagittal and the squamous suture; occipital bone: in the middle of a line between the external occipital protuberance and the point where the sagittal suture connects with the lambdoid suture. The cadavers were stored at 4 °C prior to autopsy to prevent degradation of the tissues. Following the retrieval of the tissues at room temperature, the samples were precooled at 4 °C and then kept in a − 80 °C freezer in a chemically unfixed condition until further processing. The Ethics Committee of the University of Leipzig, Germany approved the retrieval of these tissues for the given purpose (protocol number 486/16-ek). All methods were carried out in accordance with relevant guidelines and regulations. When further processed, the samples were thawed and cut to a width of 10 mm with a bone cutter (PIEZOSURGERY^®^ white, mectron s.p.a., Carasco, Italy; Fig. 1A) with a sawing blade of 0.5 mm thickness. The bone cutter automatically spills water on the blade during cutting to prevent burning of the sample while being cut. Thereafter, the samples were allocated into the following three groups: a “composite” group in which the mechanically-tested sample consisted of all three neurocranial bone layers (outer table, diploë and inner table; Fig. 1B), a “tables with diploë” group, in which the outer and inner table were separated in the middle of the diploë layer (Fig. 1C) and a “tables without diploë” group, in which the outer and inner tables were separated according to the former group, then followed by a complete removal of the diploë using sandpaper with a grit size of 60-grit to coarsely remove the diploë initially and using a 240-grit sandpaper to accurately remove the diploë close to the tables (grit sizes according to the Coated Abrasives Manufacturers’ Institute system; Fig. 1D–G). An attempt was made to separate the tables in the middle of the diploë leaving approximately 50% of the initial diploë on either table. According to the aforementioned separation procedure of the three-layered neurocranial bone the “tables with diploë” group resulted in two samples for mechanical testing (T_outer_ + D, outer table + diploë; T_inner_ + D, inner table + diploë), which both still had approximately half of the diploë attached (Fig. 2). About 0.5 mm of the diploë was removed during the layer separation with the bone cutter (value equals the thickness of the sawing blade). The “tables without diploë” group also resulted in two samples for the mechanical tests (T_outer_, outer table; T_inner_, inner table; Fig. 2). A summary of the number of samples per testing group, the retrieval site, age, post-mortem interval (PMI, time between death of the cadaver and the sample retrieval; range in this study 11–139 h) and sex ratio of the three groups is given in Table 1.

**Table 1:** The number of samples per donor for the mechanical testing groups and retrieval- and cadaver-related data are depicted.

Group	Number of samples	F	TL	TR	P	O	Number of donors	Median age (IQR) (years)	Median PMI (IQR) (h)	Female:male ratio
Composite	108	20	26	27	16	19	62	51 (39)	71 (44)	23:39
Tables with diploë	101	28	15	8	20	30	59	50 (39)	71 (43)	19:40
Tables without diploë	48	12	6	6	14	10	37	55 (40)	63 (45)	15:22

Composite, full-thickness composite group; F, frontal; O, occipital; P, parietal; PMI, post-mortem interval; IQR, interquartile range; F, frontal; O, occipital; P, parietal; TL/TR, temporal left and right.

### Mechanical testing

**Figure 3:**
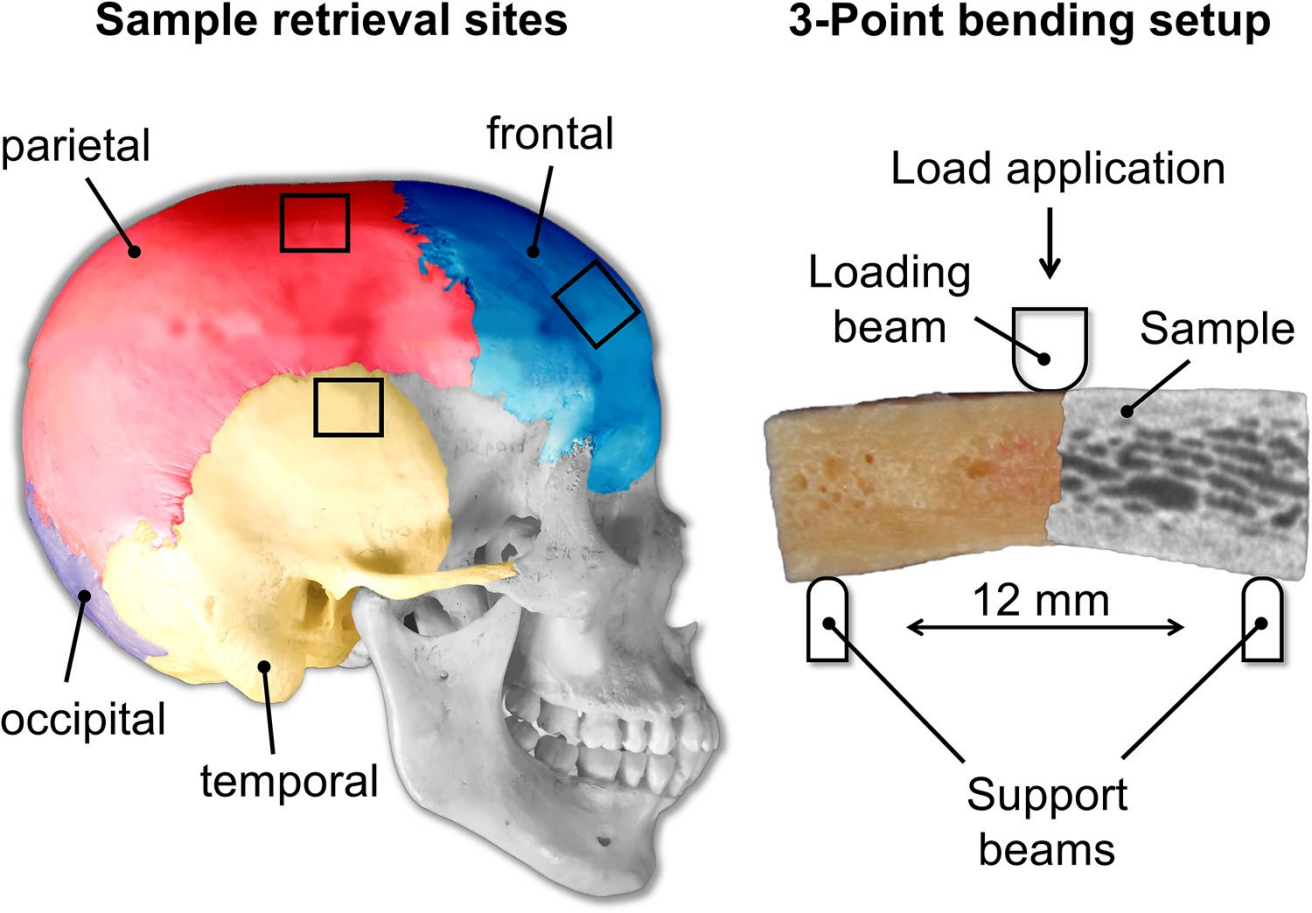
The sample retrieval sites on the neurocranium and the three-point bending setup are depicted. The black squares exemplify the sample retrieval sites for all neurocranial regions except for the occipital one. A three-layered neurocranial sample of the composite group is shown as a fusion of a bare unprocessed sample and a micro-computed tomography image of the same to illustrate the sandwich-like structure more detailed.

Prior to the mechanical testing, the thickness of each sample was determined with a digital caliper (Coolant Proof 200 mm, MeasumaX, Auckland, New Zealand; accuracy ± 0.001”). The samples were tested using a three-point bending setup on a universal testing machine (AllroundLine Table-top Z020; Zwick Roell, Ulm, Germany) equipped with an Xforce K load cell of 20 kN and testControl II measurement electronics (all Zwick Roell). The radii of the loading beam and the two support beams were 2 and 1 mm, respectively (Fig. 3). The samples were loaded until failure using a span length of 12 mm and a testing speed of 10 mm per minute. All tested samples were loaded from the scalp-facing surface to the brain-facing surface, corresponding to an in-vivo load application to the neurocranium from superficial to deep.

### Data processing and statistical analyses

Maximum force (F_max_), describing the maximum applicable force before failure of the tissue, was evaluated using the force readings from the machine. Bending strength (B_strength_) was calculated using F_max_, support span (12 mm) and measured width as well as thickness (both individual for each sample) under estimation of a bending beam with a rectangular cross-section as follows^20^:

$${\text{B}}_{{{\text{strength}}}} = \left( {3 \cdot {\text{F}}_{{\rm max} } \cdot {\text{span}}} \right)/\left( {2 \cdot {\text{width}} \cdot {\text{thickness x thickness}}} \right)$$

Excel Version 16.16 (Microsoft Corporation, Redmond, WA) and GraphPad Prism software version 8 (GraphPad Software, La Jolla, CA, USA) were used for the statistical evaluation. The Shapiro–Wilk test was used to test Gaussian distribution of the samples. Parametric data of samples were then tested using an ordinary one-way ANOVA (parametric data) or a Kruskal–Wallis test (non-parametric data). For the overall comparison of mechanical parameters between the corresponding outer and inner tables (T_outer_ + D vs. T_inner_ + D and T_outer_ vs. T_inner_) a Friedman test followed by an uncorrected Dunn’s test was applied. For a comparison of the outer and inner tables (T_outer_ + D vs. T_inner_ + D and T_outer_ vs. T_inner_) for each sub-region (frontal, temporal left and right, parietal and occipital) a two-tailed paired *t* test was applied for parametric data and a two-tailed Wilcoxon test for non-parametric data. Bivariate correlations (Pearson’s r for parametric, Spearman’s ϱ for non-parametric data) were performed between the mechanical parameters and age of the deceased, PMI and thickness of the samples. Medians and interquartile ranges (IQRs) are given in text. *p* values of 0.05 or less were considered statistically significant.

## Results

### Three-layered “full-thickness composite” group showed regional differences in maximum force but not in bending strength

**Figure 4:**
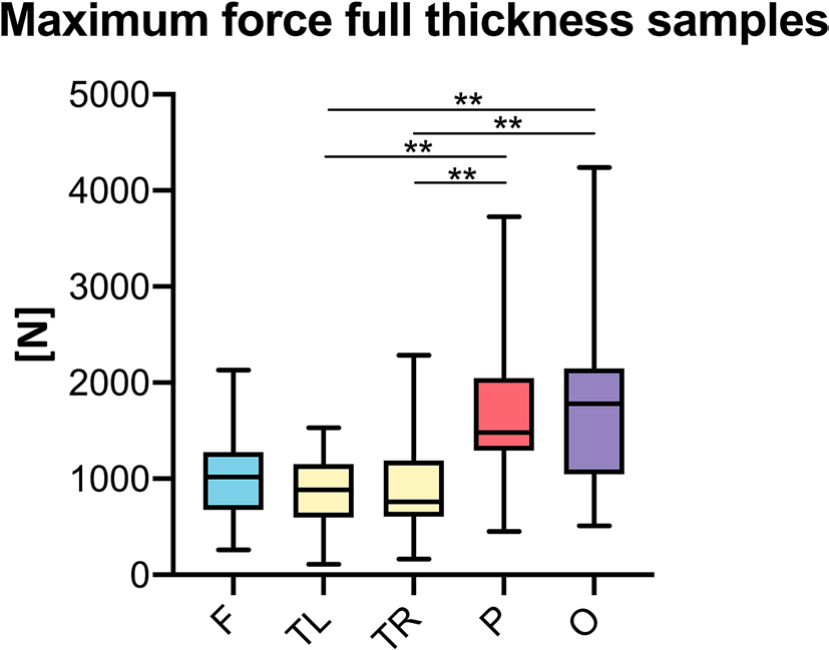
Temporal bone full-thickness composites revealed lower maximum forces compared to parietal and occipital samples. F, frontal; O, occipital; P, parietal; TL/TR, temporal left/right; **p < 0.01.

When comparing the complete bone composites among the five investigated regions, the left (886 N, IQR = 555 N) and right (763 N, IQR = 583 N) temporal bone samples showed a significantly lower F_max_ compared to the parietal (1479 N, IQR = 757 N; both *p* = 0.002) and occipital (1781 N, IQR = 1099 N, left temporal: *p* = 0.003; right temporal: *p* = 0.004) samples (Fig. 4). There were no significant differences between frontal and temporal composites nor side-dependent differences for left and right temporal samples. Intact bones were similar and statistically non-different regarding their B_strength_. A summary of the mechanical values for these regions is given in Table 2.

**Table 2:** Summarized mechanical parameters.

	Pooled		Frontal		Temporal left		Temporal right		Parietal		Occipital	
F_max_ Composite (N)	1054 (810)		1019 (602)		886 (555)		763 (583)		1479 (757)		1781 (1099)	
F_max_ T_outer_ + D | T_inner_ + D (N)	339 (275)	206 (206)	342 (309)	241 (217)	209 (144)	145 (122)	381 (185)	167 (250)	430 (361)	284 (198)	339 (318)	151 (227)
F_max_ T_outer_ | T_inner_ (N)	264 (260)	132 (143)	259 (251)	124 (181)	196 (151)	71 (47)	147 (178)	111 (117)	377 (301)	171 (137)	360 (440)	141 (200)
B_strength_ Composite [MPa]	67 (45)		70 (71)		72 (45)		78 (45)		59 (35)		59 (39)	
B_strength_ T_outer_ + D | T_inner_ + D [MPa]	60 (42)	59 (37)	56 (50)	63 (52)	55 (36)	59 (27)	73 (34)	59 (38)	70 (76)	56 (46)	56 (36)	58 (46)
B_strength_ T_outer_ | T_inner_ [MPa]	92 (52)	90 (53)	99 (64)	91 (58)	107 (73)	111 (38)	102 (130)	119 (149)	98 (40)	78 (54)	77 (43)	68 (58)

F_max_, maximum force; T_outer_ + D, outer table + diploë; T_inner_ + D, inner table + diploë; T_outer_, outer table and T_inner_, inner table; Median, interquartile ranges are given in parentheses.

### The “tables without diploë” group revealed significantly different maximum forces between outer and inner tables as well as different sites of the neurocranium, but showed similar bending strengths

When all of the five regions of the neurocranium were pooled, T_outer_ + D showed a significantly higher F_max_ (median 339 N, IQR = 275 N) compared to T_inner_ + D (median 206 N, IQR = 206 N, *p* = 0.011), but both pooled sample cohorts were statistically non-different regarding their B_strength_. When each region was evaluated independently F_max_ of T_outer_ + D was also significantly higher compared to T_inner_ + D (frontal: *p* = 0.010; temporal left: *p* = 0.011; temporal right: *p* = 0.029; parietal: *p* < 0.001; occipital: *p* = 0.001). The F_max_ comparison of T_outer_ + D between regions revealed a significantly higher value for parietal samples (median 430 N, IQR = 361 N) compared to the left temporal samples (median 209 N, IQR = 144 N, *p* = 0.010). None of the remaining mechanical parameters differed between the regions on a statistically significant level. Moreover, B_strength_ was similar and statistically non-different in each region in line with the pooled samples. A summary of the obtained mechanical values for this group is given in Table 2.

### The “tables without diploë” group showed stronger outer compared to inner tables and weaker temporal regions compared to the remaining neurocranial bone locations, but similar bending strengths

When pooling the data of all regions, T_outer_ revealed a significantly higher F_max_ (median 264 N, IQR = 260 N) compared to T_inner_ (median 132 N, IQR = 143 N, *p* = 0.003), but was statistically non-different regarding its B_strength_. The F_max_ of T_outer_ was significantly higher compared to T_inner_ in the frontal (median T_outer_ = 259 N, IQR = 251 N, median T_inner_ = 124 N, IQR = 181 N, *p* < 0.001), parietal (median T_outer_ = 377 N, IQR = 301 N, median T_inner_ = 171 N, IQR = 137 N, *p* = 0.002) and occipital (median T_outer_ = 360 N, IQR = 440 N, median T_inner_ = 141 N, IQR = 200 N, *p* = 0.013) regions, but non-different in both temporal regions. The F_max_ comparison of T_outer_ between regions revealed significantly lower values for both left (median 196 N, IQR = 151 N, *p* = 0.023) and right temporal (median 147 N, IQR = 178 N, *p* = 0.012) samples compared to parietal ones (median 377 N, IQR = 301 N). None of the remaining mechanical parameters differed between the regions on a statistically significant level. B_strength_ was statistically non-different when comparing each region individually. A summary of the obtained mechanical values for this group is given in Table 2.

### Comparison of mechanical parameters between groups

**Figure 5:**
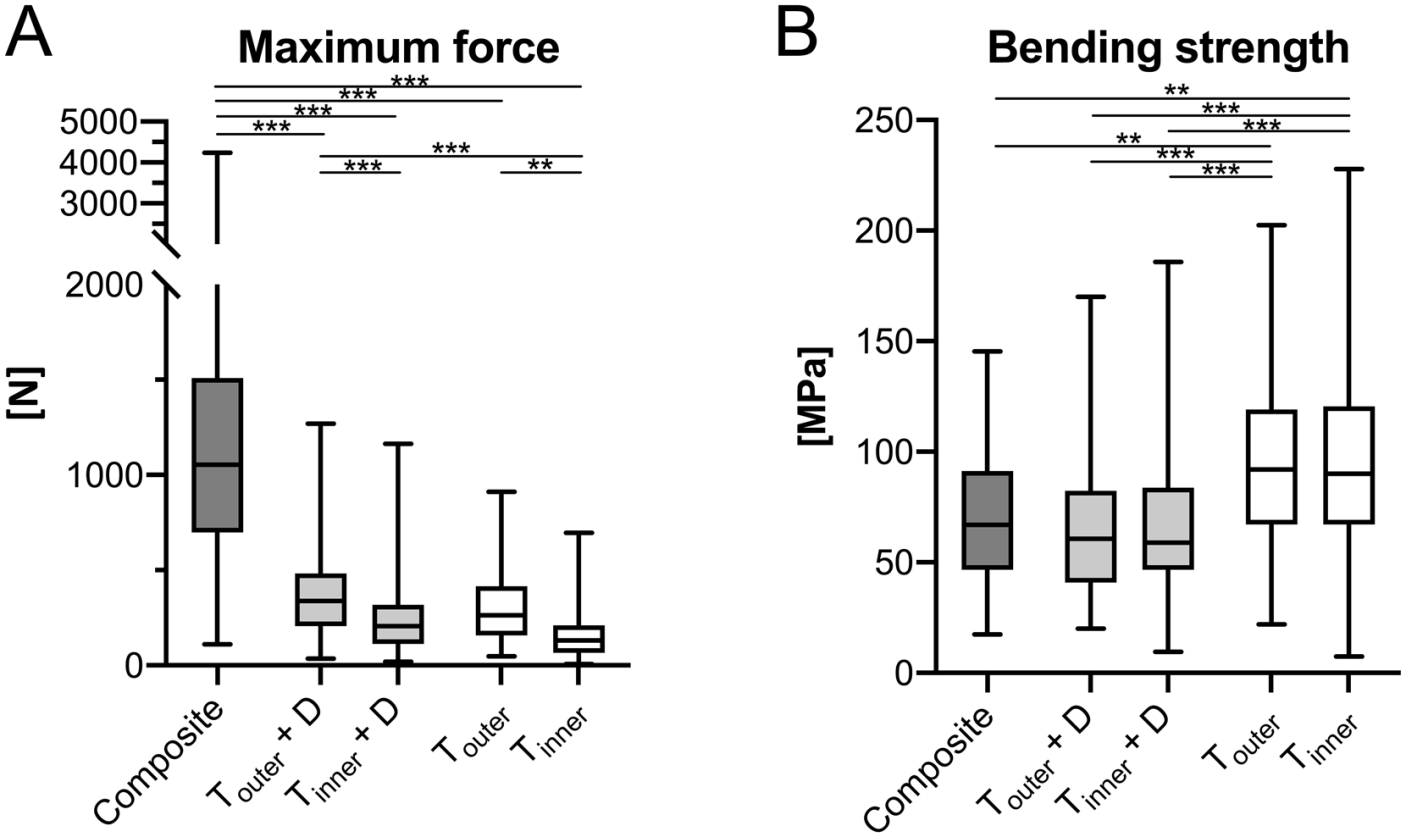
The maximum force (**A**) and bending strength (**B**) are depicted for the different groups. Composite, full-thickness group; T_outer_ + D, outer table + diploë; T_inner_ + D, inner table + diploë; T_outer_, outer table; T_inner_, inner table; **p < 0.01; ***p < 0.001.

**Figure 6:**
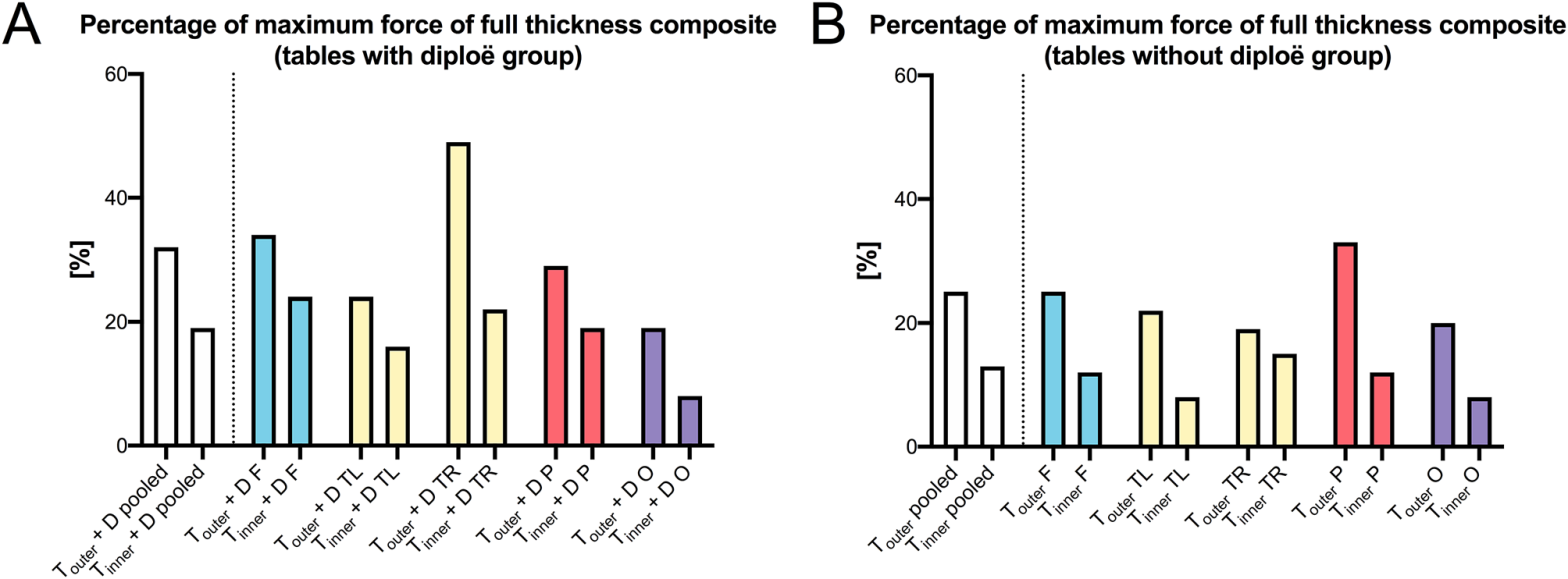
The maximum forces of (**A**) the “tables with diploë” group and the (**B**) “tables without diploë” group are given as a percentage of the “composite” group with the latter representing 100%. T_outer_ + D, outer table + diploë; T_inner_ + D, inner table + diploë; T_outer_, outer table and T_inner_, inner table; F, frontal; O, occipital; P, parietal; TL/TR, temporal left and right.

The F_max_ values of the pooled group (median 1054 N, IQR = 810 N) were significantly higher compared to both the samples of the separated group (T_outer_ + D: median 339 N, IQR = 275 N, *p* < 0.001; T_inner_ + D: median 206 N, IQR = 206 N, *p* < 0.001) as well as the samples of the separated and removed group (T_outer_: median 264 N, IQR = 260 N, *p* < 0.001; T_inner_: median 132 N, IQR = 143 N, *p* < 0.001; Fig. 5A). Neither the F_max_ values of T_outer_ + D nor the one of T_inner_ + D were significantly different from the groups, in which the diploë was removed (Fig. 5A). While the F_max_ values for the T_outer_ + D group were statistically higher compared to T_inner_ (*p* < 0.001), the T_inner_ + D group was statistically non-different from the T_outer_ group (Fig. 5A). The B_strength_ of the composite group statistically differed from both the B_strength_ of both layers of the separated and removed group (T_outer_, *p* = 0.003; T_inner_, *p* = 0.004; Fig. 5B). The B_strength_ of the T_outer_ + D layer of the separated only group (median 60 MPa, IQR = 42 MPa) differed from both layers of the separated and removed group (median T_outer_ = 92 MPa, IQR = 52 MPa, *p* < 0.001; median T_inner_ = 90 MPa, IQR = 53 MPa, *p* < 0.001; Fig. 5B). Equally, the B_strength_ of the T_inner_ + D layer of the separated only group (median 59 MPa, IQR = 37 MPa) differed from both layers of the separated and removed group (T_outer_: *p* < 0.001; T_inner_: *p* < 0.001) on a statistically significant level (Fig. 5B). When related to the F_max_, which the three-layered composite of the respective area withstood, T_outer_ + D reached between 19% (occipital) and 49% (temporal right) of this force (Fig. 6A). T_inner_ + D only withstood between 8% (occipital) and 24% (frontal) of the F_max_ of the three-layered composite (Fig. 6A). Similarly, when related to the F_max_, which the three-layered composite of the respective area withstood, T_outer_ reached between 19% (temporal right) and 33% (parietal) of this force (Fig. 6B). T_inner_ only withstood between 8% (temporal left and occipital) and 15% (temporal right) of the F_max_ of the three-layered composite (Fig. 6B).

### Age-, PMI-, sex-, and thickness correlations

**Figure 7:**
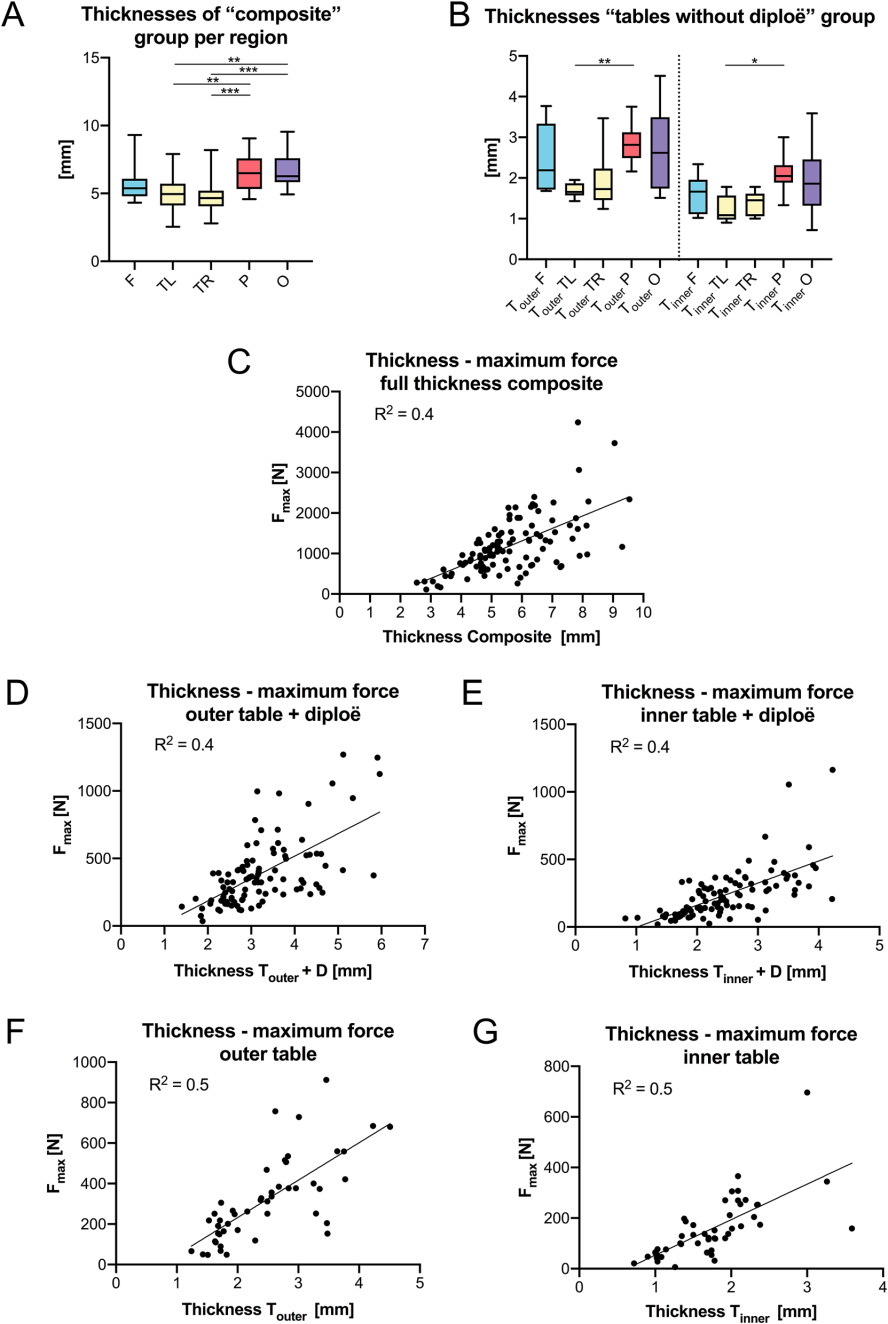
The mechanical parameters of neurocranial bone regions are depicted in relation to the thickness of the tested sample. (**A**) full-thickness “composite” group per region, (**B**) “tables without diploë” group, (**C**) correlation thickness—maximum force (F_max_) of “composite” group, (**D**) correlation thickness—F_max_ outer table “tables with diploë” group (T_outer_ + D), (**E**) correlation thickness—F_max_ inner table “tables with diploë” group (T_inner_ + D), (**F**) correlation thickness—F_max_ outer table (T_outer_) “tables without diploë” group, (**G**) correlation thickness—F_max_ inner table (T_inner_) “tables without diploë” group; F, frontal; O, occipital; P, parietal; TL/TR, temporal left and right; *p < 0.05; **p < 0.01; ***p < 0.001.

Both left and right temporal full-thickness composites were significantly thinner compared to parietal (temporal left: *p* = 0.004, temporal right: *p* < 0.001) and occipital (temporal left: *p* = 0.005, temporal right: *p* < 0.001) composites (Fig. 7A). Both T_outer_ + D (*p* < 0.001) and T_inner_ + D (*p* < 0.001) were significantly thicker compared to the separated and diploë-removed group. For left temporal samples, both T_outer_ and T_inner_ were significantly thinner compared to the parietal (T_outer_: *p* = 0.006; T_inner_: *p* = 0.017) region (Fig. 7B). With the samples of all regions pooled, T_outer_ was significantly thicker compared to T_inner_ (*p* < 0.001). However, when observing those regions individually, this difference was only observed for frontal (*p* = 0.001), parietal (*p* < 0.001) and occipital (*p* = 0.017) samples. The thickness values of the tested samples are depicted in Table 3.

**Table 3:** The thicknesses of the tested samples are depicted separated per region.

	Pooled		Frontal		Temporal left		Temporal right		Parietal		Occipital	
Thickness Composite (mm)	5.4 (1.8)		5.4 (1.3)		5.0 (1.6)		4.7 (1.1)		6.5 (2.3)		6.3 (1.8)	
Thickness T_outer_ + D | T_inner_ + D (mm)	3.1 (1.2)	2.4 (1.0)	3.2 (1.7)	2.4 (1.4)	2.6 (0.3)	2.0 (0.7)	2.9 (1.0)	2.4 (1.0)	3.2 (1.3)	2.6 (1.2)	3.2 (1.4)	2.5 (1.0)
Thickness T_outer_ | T_inner_ (mm)	2.4 (1.3)	1.7 (0.8)	2.2 (1.6)	1.7 (0.8)	1.7 (0.3)	1.1 (0.6)	1.7 (0.8)	1.5 (0.6)	2.8 (0.6)	2.1 (0.4)	2.6 (1.8)	1.9 (1.1)

T_outer_ + D, outer table + diploë; T_inner_ + D, inner table + diploë; T_outer_, outer table and T_inner_, inner table; Interquartile ranges are given in parentheses.

F_max_ of all groups showed a significant moderate to strong positive and linear correlation with the thickness of the samples (composite: r = 0.624, *p* < 0.001, Fig. 7C; T_outer_ + D: r = 0.602, *p* < 0.001, Fig. 7D; T_inner_ + D: r = 0.705, *p* < 0.001, Fig. 7E; T_outer_: r = 0.769, *p* < 0.001, Fig. 7F; T_inner_: r = 0.789, *p* < 0.001, Fig. 7G). The sample thickness of the composite group (r = 0.243, *p* = 0.011) and T_inner_ + D (r = 0.214, *p* = 0.032) samples of the separated only group showed a weak positive correlation with age. B_strength_ of T_inner_ was the only mechanical parameter which significantly correlated with PMI (r = 0.302, *p* = 0.037). All mechanical parameters obtained in this study were independent of the sex of the cadaver irrespective of the tested subgroup. Apart from the weak negative correlation between B_strength_ of the composite group (r = -0.285, *p* = 0.003), the mechanical parameters in this study were also independent of age.

## Discussion

Mechanical properties of the human neurocranium have so far been obtained using three-point bending^6,8,10,14–17^, four-point bending^36^, compressive^7,11,13,25^, tensile^7,13,16,19,22,24^ and shear^7^ test protocols, as well as ultrasonic pulse transmission techniques^21^. The here presented study for the first time systematically investigated the contribution of the individual bone layers of the neurocranium to the mechanical behavior of the three-layered composite involving all major flat bones of the neurocranium in a large sample size over a broad age range. Overall, the thickness of the samples correlated with the applicable F_max_ irrespective of the tested group in this given study. Temporal bone samples were significantly thinner and withstood lower loads compared to the parietal and occipital regions. Similarly, the T_outer_ only revealed higher failure loads compared to T_inner_ when being thicker at the same time, which was true for the frontal, parietal and occipital samples, but not for the temporal samples of similar thickness. An exception to this ‘thicker bone—stronger bone’ relation was the T_outer_ of the right temporal region, which showed a significantly lower F_max_ value compared to the parietal region despite being of a similar thickness. This finding might be explained by the limited sample sizes in this study, with only six T_outer_ samples for the subgroup at the right temporal region, which likely caused a statistical type I error. Lower sample sizes are prone to be biased by outliers that show, e.g., extremes, such as low mechanical resistance of a tested bone sample due to a decreased bone density^10^ or conditions that negatively affect the bone quality such as Paget’s disease^37^, referring to unknown conditions as pre-existing bone diseases were used as ultimate exclusion criteria during sampling. With regards to the diploë-table ratio of the neurocranium, two important observations were made in the given study. Firstly, the T_inner_ thickness was statistically non-different between all investigated regions. Secondly, the T_outer_ thickness was statistically non-different between the different neurocranial regions investigated in this study apart from the left temporal T_outer_, which was thinner than the parietal site. Based on these observations and the assumption that the divergent value is biased, the here presented findings indicate that the thickness of the three-layered neurocranium is mainly determined by the thickness of the diploë rather than the outer or inner table. The covariation between diploë and cranial thickness is supported by a former radiographic study on 256 neurocranium samples measured on frontal, occipital and left and right euryon^38^. Temporal bones have a comparatively low amount of diploë^4^, which diminishes towards the inferior portion of the bone^39^. The results of this study showed that the intact temporal samples showed significantly lower loads compared to frontal, parietal and occipital samples, which supports the hypothesis that the diploë thickness is of high biomechanical importance when human neurocranium is simulated in computer models^38^.

### The individual outer and inner tables only reach 25% and 13% of the maximum forces of the full-thickness composite

The individual layer tests in this study revealed that the mechanical characteristics of the human neurocranium are based on the arrangement of the three layers and their mutual connection rather than being a summative of the load resistance of the individual layers. When all samples were pooled T_outer_ and T_inner_ reached only 25% and 13% of the F_max_ value of the full-thickness composite. Cancellous bone has a lower compressive strength compared to compact bone in general^13^, and, therefore, the bare material properties of the diploë are insufficient to explain vastly higher load resistance of the intact neurocranial composite compared to the individual layers. The overall arrangement of the human neurocranium well corresponds to a special class of engineering materials—the sandwich-structured composite—with two thin but strong skin sheets and a lightweight but thick core connecting the strong skin sheets. This type of engineering composite with a core of a material with a lower strength provides an overall high bending stiffness and high bending strength with a much lower density compared to full thickness samples of the strong sheet material. In line with this, the “tables with diploë” group, in which approximately half of the diploë remained attached to the T_outer_ and T_inner_ was mechanically indifferent from the group, in which the diploë was removed. Taking into account the complex trabecular orientation within the diploë without a direct connection of T_outer_ and T_inner_ via trabeculae perpendicular to the surface of the tables, we hypothesize that the loads that are applied to the T_outer_ from external in case of head impacts are dissipated via the diploic trabeculae to eventually act on larger areas on the T_inner_ compared to the area of impact on the T_outer_. Based on this load dissipation principle between the two tables, less load acts on the T_inner_ per area compared to the T_outer_, but the area this load is dissipated to via the trabeculae should be larger. Therefore, it is plausible that, in vivo, the T_inner_ is sufficiently load-resistant compared to the T_outer_ even though being thinner, which provides as a biomechanical explanation for the thickness differences between the two layers. An alternative hypothesis of the observed thickness difference between the two tables is the exposure of the T_outer_ to muscular loads, which are comparatively higher than intracerebral loads acting on the T_inner_^21^, naturally omitting the necessity for a thicker T_inner_. Despite containing significantly thicker samples, the “tables with diploë” group was statistically non-different compared to the “tables without diploë” group from a (bio)-mechanical perspective. These findings indicate that bone trabeculae require the respective second cortical table to effectively dissipate loads, likely to larger surface as described above or being able to store energy by being compressed between the two tables, while at the same time minimizing the weight of the bone composite.

The B_strength_ in this study was similar and statistically non-different between the here investigated sites within one testing group from the various regions or when comparing the corresponding outer and inner tables within the groups with and without diploë, respectively. However, the outer and inner tables of the “tables with diploë” group revealed a significantly lower B_strength_ compared with the tables, for which the cancellous bone was additionally removed. This is explained by the fact that the tables without attached diploë were significantly thinner compared to the ones with an attached diploë, but non-different in F_max_ resistance at the same time. Significant thickness differences when comparing two materials are critically influencing the obtained B_strength_ as the thickness is reflected in the B_strength_ equation as a squared divisor^20^. Consequently, the compact T_outer_ and T_inner_ show a higher B_strength_ compared to the composite of the compact table with an attached similarly composed^40^ but more porous^41^ and weaker^13^ diploë layer that is adding significantly to the thickness of the sample, but not to its mechanical strength. The here reported B_strength_ of 67 MPa is similar to the values of 85 MPa obtained from frontal and parietal regions of eight fresh-frozen cadavers using a testing velocity of 30,000 mm/min^12^ and the 64 to 86 MPa obtained from 114 unembalmed fronto-parietal samples using a testing speed of 0.06 mm/min^10^. A study involving Crosado-embalmed^42^ cadavers using an identical testing velocity as in the given study of 10 mm/min reported a lower B_strength_ of 42 MPa and 53 MPa for the two investigated human neurocrania^6^, likely due to an embrittlement of the tissue following the chemical treatment or a statistical bias caused by the low sample size in the former work. The B_strength_ of the composite group in this given study decreased with age, presumably caused by the concomitant age-related thickening of the samples without a concurrent increase of F_max_ values. However, it should be noted that the found negative correlation between B_strength_ and age revealed a limited “statistical strength” as the respective r value was low. The age-related thickening of the neurocranium is likely caused by a thickening of the diploë rather than the tables as thickening with age was seen in the separated group, but not in the separated and diploë-removed group in this study. The influence of age on the mechanical behavior of the neurocranium can be deemed vague rather than contradictory. Some authors report that mechanical parameters are independent of age^17,19^, whereas others detected age-related increases of elasticity^14,18^ and compressive strength^18^, but decreases of fracture loads^43^. Regarding the former, it must be considered that generally limited sample sizes and restricted age ranges might be insufficient to detect age-related mechanical changes caused by small effect sizes, which are simultaneously strongly affected by the other parameters such as sample thickness or the load application vector with respect to the anisotropic bone. The weak positive correlation between B_strength_ and PMI of T_inner_ might have been caused by an increased collagen cross-linking post-mortem or by handling- and storage-related dehydration processes after death. As the load resistance of the here tested samples was not decreasing as a sign of tissue degradation in general, it is concluded that cadaveric bone retrieved during forensic autopsies can be used for the purposes, when cadavers or samples are kept cool constantly. Material properties of native bones are paramount to fabricate lifelike physical surrogates for surgical^44^ or forensic applications^9,45^. Moreover, material properties of the neurocranium are applied in computational simulations of the human head to simulate various head impact scenarios^3^. While this given study focused on the mechanical properties of human neurocranium only, it has to be noted that surrounding soft tissues such as the periosteum or the dura mater might be of importance to replicate the response of the human head to impact forces in a realistic manner^9,46^.

### Limitations

Firstly, the given study is limited in sample size for each subgroup in spite of the large overall number of samples, which might have affected the here stated results via multiple group comparisons. However, robust post hoc tests were used for statistical analyses. Secondly, the bone samples are naturally convex towards the outer surface and, therefore, the bending stress assuming a straight beam with a rectangular cross-section represents an oversimplification, which unpreventably affected the results. Thirdly, the diploë removal might have been incomplete which could have influenced the here stated mechanical parameters. Fourthly, even though the specimens in this study were cut using a high-quality bone cutter that is certified to be used in clinical routine, the resulting dimensions minutely differed, which could have affected the given results. Fifthly, even though an attempt was made to separate the two tables in the middle of the diploë layer, this could not be achieved in every single sample due to the convex geometry of the neurocranial towards the outer surface. Sixthly, shear forces likely occurred due to the setup of this study and the structure of the tested tissue. This might have affected the measured B_strength_ in this study. Seventhly, this given study did not determine the individual bone densities that are influenced by various conditions such as osteoporosis, which reflects on the bone’s mechanical strength^47^. Hence, the here reported statistical comparisons between the individual groups might have been biased by differences in bone densities. Lastly, all human neurocranial samples show a complex three-dimensionally curved geometry. As the sample curvature was not measured for each individual sample of this study, its potential influence on the here reported biomechanical parameters remains unknown.

## Conclusion

The thicknesses of bones of the neurocranium critically influence their load-deformation properties. This study provides evidence that the neurocranial thickness is predominantly determined by the diploë, which thickens with age. The three-layered composite is up to four and eight times more load resistant than the individual outer and inner tables, respectively. Presuming storage of the cadaver at 4 °C at the earliest possible point after death, neurocranial samples retrieved during autopsy are suitable for mechanical testing purposes for at least five days post-mortem.

## Data Availability

The datasets generated during and/or analyzed during the current study are available from the corresponding author on reasonable request.
